# Expression of Concern for The pseudokinase tribbles homolog 3 interacts with ATF4 to negatively regulate insulin exocytosis in human and mouse β cells

**DOI:** 10.1172/JCI213205

**Published:** 2026-10-15

**Authors:** Chong Wee Liew, Jacek Bochenski, Dan Kawamori, Jiang Hu, Colin A. Leech, Krzysztof Wanic, Maciej Malecki, James H. Warram, Ling Qi, Andrzej S. Krolewski, Rohit N. Kulkarni

Original citation: *J Clin Invest*. 2010;120(8):2876–2888. https://doi.org/10.1172/JCI36849

Citation for this expression of concern: *J Clin Invest*. 2026;136(20):e213205. https://doi.org/10.1172/JCI213205

The authors recently alerted the Editors to errors in the immunoblot data presented in Figure 3E, Figure 5F, and Supplemental Figure 3A, which included duplicated images that were used to represent distinct samples. Although the authors indicated that they have original data to correct these panels, given the number of errors and extensive time that has elapsed since publication, the Editors have requested institutional oversight into this matter and will alert readers to the outcome when the institutional evaluation is complete.

## Version 1 10/15/2026

Electronic publication

